# Supplementary material for: Massive perturbation of sound representations by anesthesia in the auditory brainstem
Source: Sci Adv. 2024 Oct 18;10(42):eado2291. doi: 10.1126/sciadv.ado2291 (PMC11488538; doi:10.1126/sciadv.ado2291)
Supplement: Supplementary file 1 — Figs. S1 to S6 [file sciadv.ado2291_sm.pdf]

Science Advances
AAAS

Supplementary Materials for

Massive perturbation of sound representations by anesthesia in the auditory brainstem

Etienne Gosselin *et al.*

Corresponding author: Brice Bathellier, brice.bathellier@cnrs.fr

Sci. Adv. **10**, eado2291 (2024)
DOI: 10.1126/sciadv.ado2291

This PDF file includes:

Figs. S1 to S6

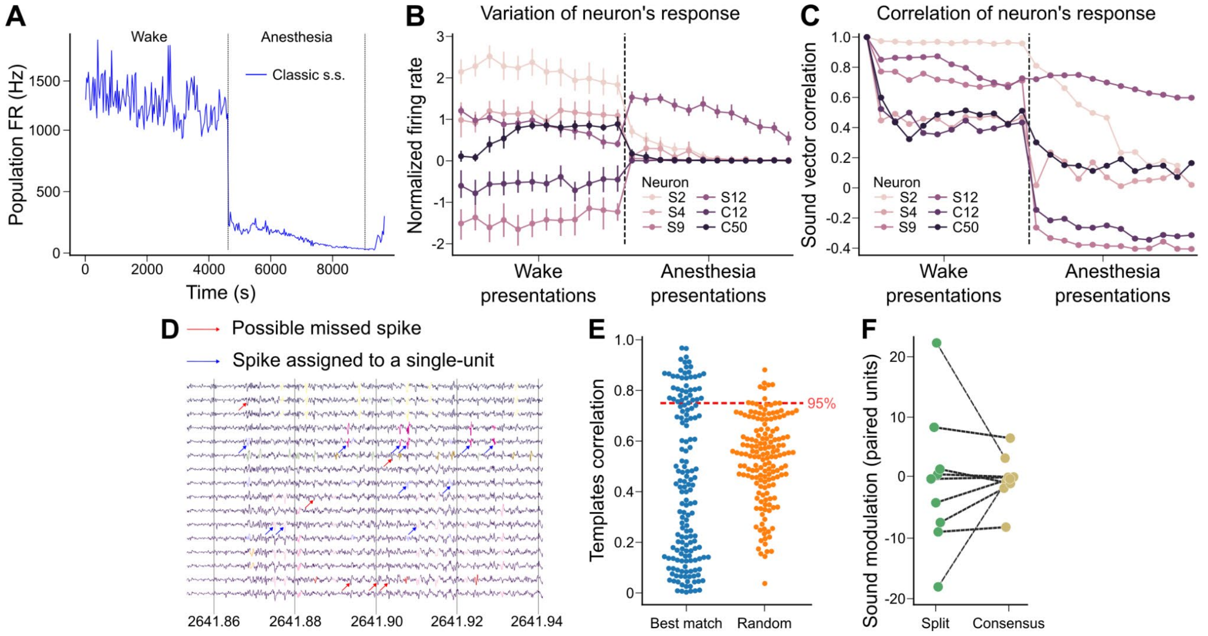

Supplementary Fig. 1: Anesthesia induces a massive and sudden modification of single neurons response

A. Summed firing rate across time for all neurons clustered with the classic spike-sorting strategy. **B.** Variation of time-averaged firing rate for 6 example neurons (paired neurons 2, 4, ,9 and 12 from the split s.s. dataset, and neurons 12 and 50 from the consensus s.s. dataset) to their 10 most responsive sounds, normalized by the mode (maximum - minimum response) across wake and anesthetized presentations. **C.** Correlation of responses to all sounds between the first wake and all sound presentations for 6 example neurons. **D.** Phy GUI visualization of a snippet of awake recording, showing successfully assigned and possibly missed spikes. **E.** Correlation of templates for the best matching neurons between Split s.s. and Consensus s.s. neurons from the same experiments during Wake (blue), and random distribution of best matching templates of neurons from different experiments (orange). The threshold of significance of matches is shown in red. **F.** Evoked activity of 9 neurons with matching templates during Wake for Split and Consensus spike sorting. The matching waveforms had to pass the significance threshold shown in E and a second criterion imposing that no other template closely resembles the matched template (no secondary match at a distance of <0.2 correlation, see Methods).

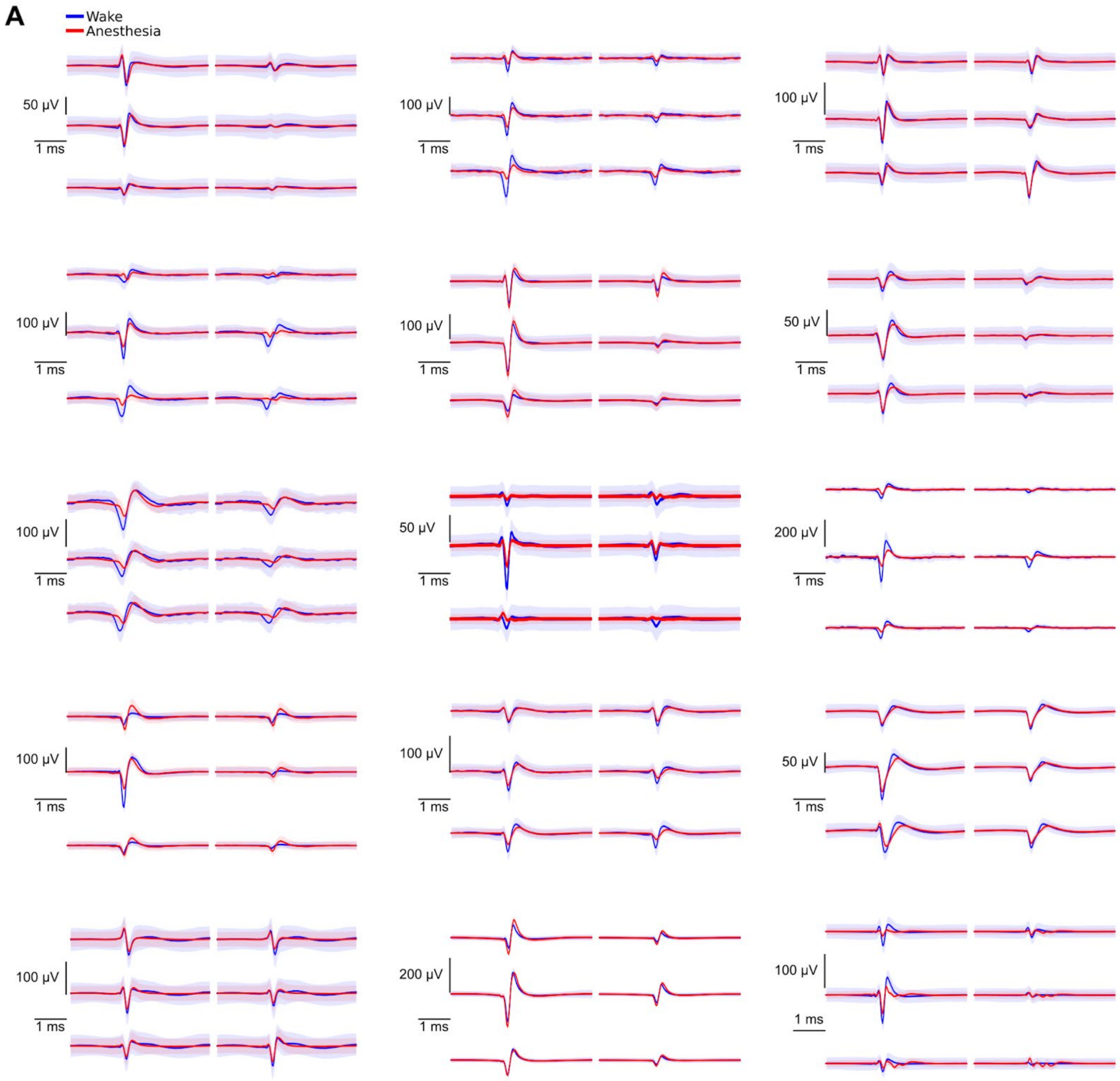

Fig. S2.: Waveform similarity across anesthetized and awake states for matched units after split spike sorting

A. Spike waveforms for 15 matched neurons of the split s.s. dataset for 6 channels centered around the maximal amplitude channel of each neuron.

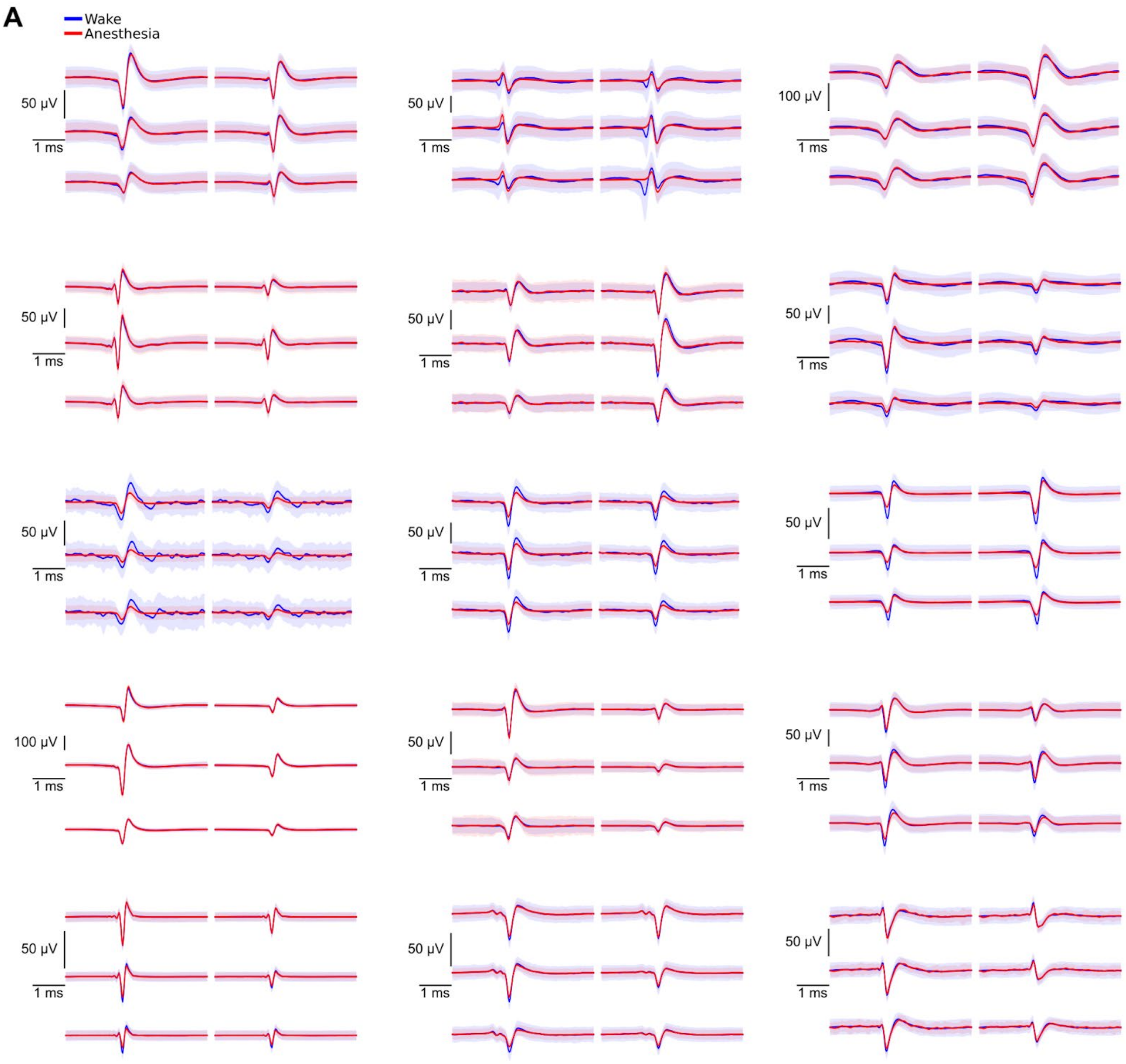

Fig. S3.: Waveform similarity across anesthetized and awake states (consensus spike sorting)

A. Spike waveforms for 15 matched neurons of the consensus spike sorting dataset for 6 channels centered around the maximal amplitude channel of each single unit.

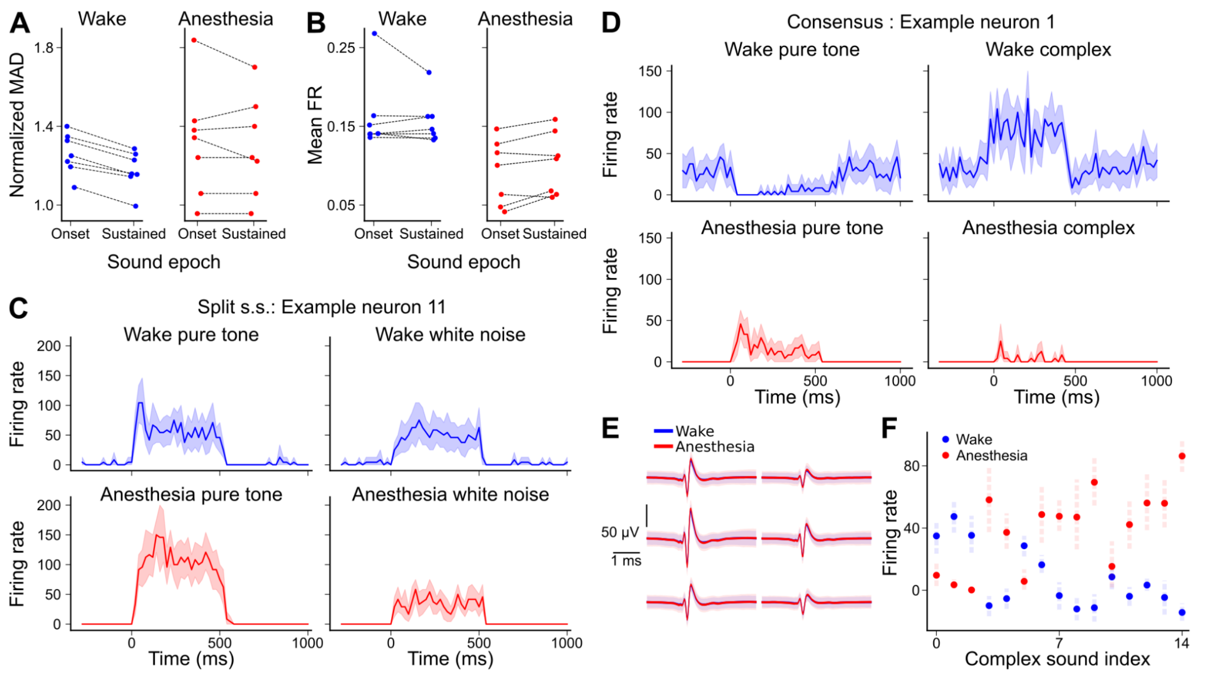

Supplementary Figure 4: A fraction of sound onset spikes are missed during spike-sorting, which does not systematically bias recorded responses

A. Median Absolute Deviation (MAD) of voltage traces during Onset and Sustained response, normalized by the MAD for 100 ms before sound onset. **B.** Mean firing rate of all neurons at Onset (50 ms after sound onset) and during Sustained response (50 to 150 ms after sound onset) for all sounds and all experiments. **C.** PSTH of response of a split s.s. example neuron to one pure tone and one white noise during both Wake and Anesthesia. Onset firing rate is visibly not higher than sustained response, although a primary-like response type is expected from this unit. **D.** PSTH of response of a consensus s.s. example neuron to one pure tone and one complex sound during both Wake and Anesthesia. **E.** Spike waveforms of the neuron presented in **D** during Wake and Anesthesia. **F.** Time-averaged firing rate of the neuron presented in **D** in response to all presentations of the 14 complex sounds at 70 dB SPL during Wake and Anesthesia.

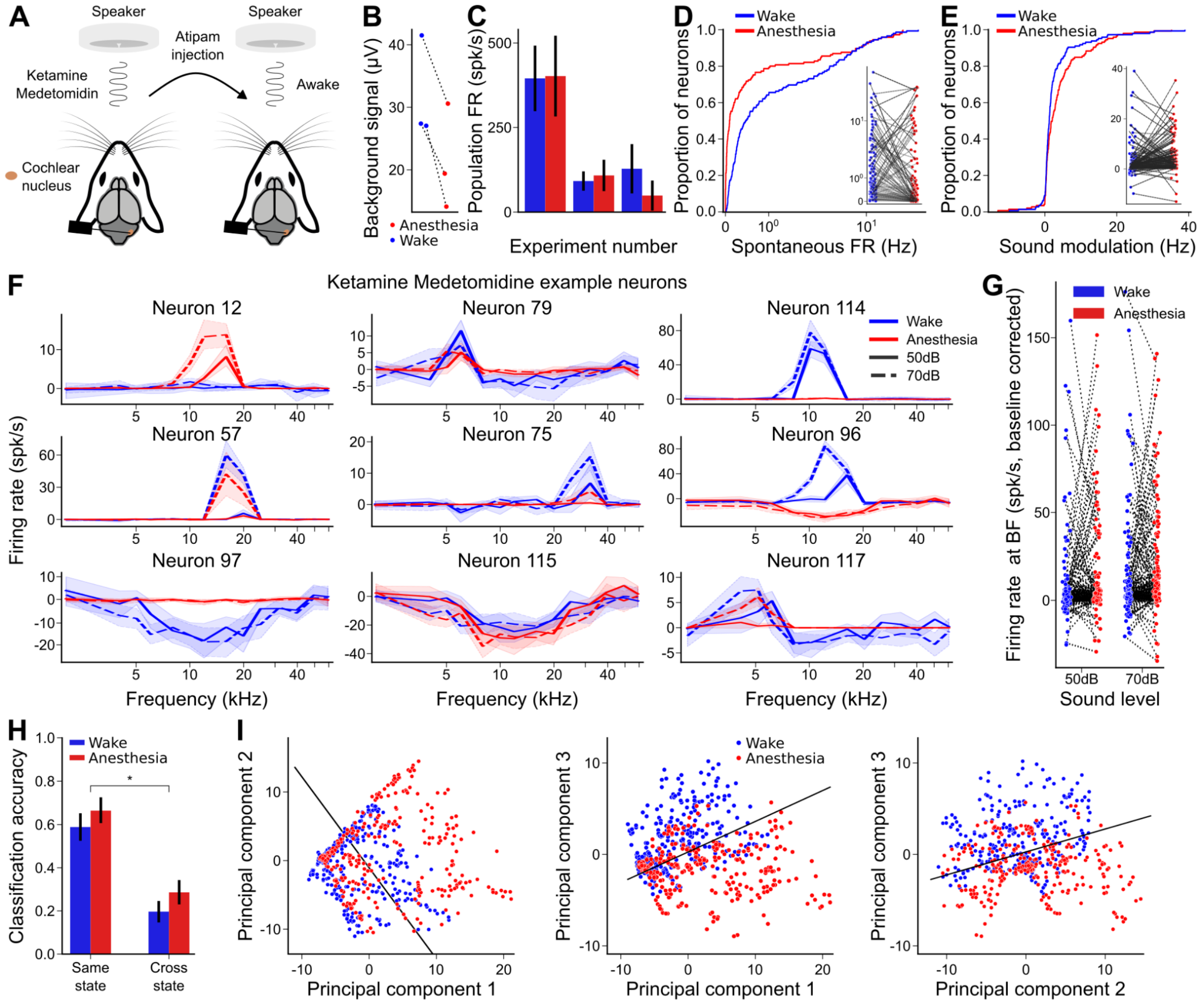

Supplementary Figure 5: Ketamine-Medetomidine anesthesia has a weaker, yet significant impact on sound encoding in the cochlear nucleus

A. Schematic drawing of the time-reversed experiment performed with Ketamine-Medetomidine. **B.** Summed firing rate of neurons across time clustered with a classic spike-sorting strategy. **C.** Time-averaged, summed population firing rate during Anesthesia and Wake in the 3 recordings. **D.** Cumulative distribution plot of spontaneous firing rate of neurons during Anesthesia and Wake. Paired values in both states are shown in inset. **E.** Cumulative distribution plot of baseline-corrected, sound-evoked firing rate of neurons during Anesthesia and Wake. Paired values in both states are shown in inset. **F.** Pure tone tuning curves for 9 single units across states (awake = blue and anesthetized = red). The dashed and solid lines represent the tuning at 50 and 70 dB SPL respectively. **G.** Baseline-corrected firing rate for neurons at their best frequency during both states. **H.** Barplots of same state and cross state decoding accuracy of all sounds for non time-averaged responses (same-state Wake accuracy = 0.58±0.02, cross-state Wake accuracy = 0.23±0.02, same-state Anesthesia accuracy = 0.73±0.02, cross-state Anesthesia accuracy = 0.29±0.02, N = 307 sounds). Decoding accuracy is significantly higher in both states for same-state classification than cross-state classification (Wake p-value = $1e^{-33}$, Anesthesia p-value = $1e^{-39}$, Wilcoxon signed-rank test, N = 307 sounds). **I.** Distribution of sound responses during wake (blue) and anesthesia (red) after projection into the first three principal components for KM dataset. The black line indicates the plane of best separation between the two states as defined by linear support vector machine classification.

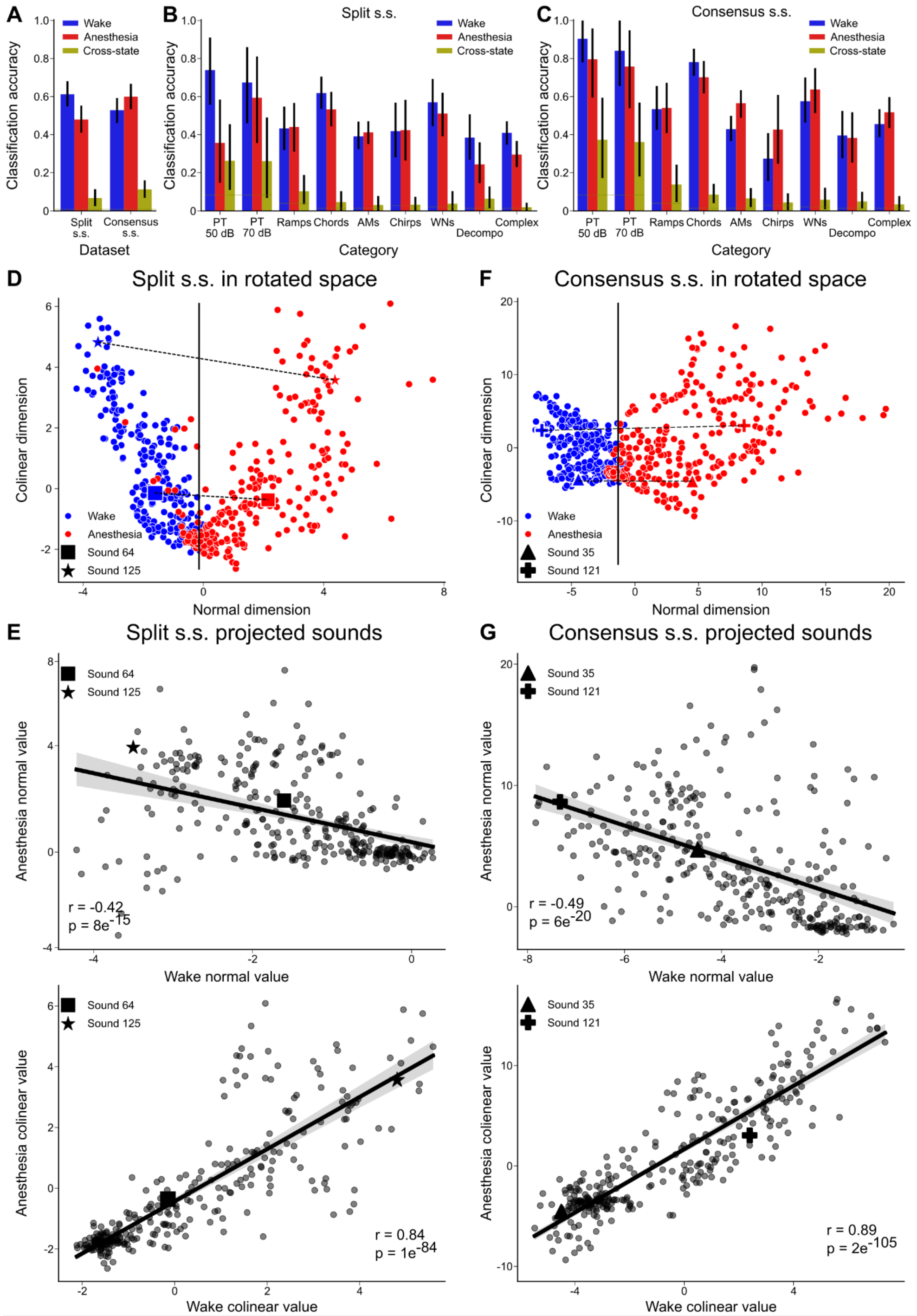

Supplementary Figure 6: The transformation of sound representation across isoflurane anesthesia and wakefulness preserves the structure of the representation space.

A. Barplots of same-state and cross-state decoding accuracy for awake and isoflurane datasets for all sounds at 70 dB SPL or more (Split s.s. dataset: same-state Wake accuracy = 0.60±0.03, Anesthesia accuracy = 0.59±0.03, cross-state accuracy = 0.07±0.01, Wilcoxon signed-rank test of classification, p = $1e^{-30}$. Consensus s.s. dataset: same-state Wake accuracy = 0.53±0.02, Anesthesia accuracy = 0.51±0.03, cross-state accuracy = 0.11±0.02, Wilcoxon signed-rank test of classification, p = $2e^{-27}$). **B,C.** Barplots of same-state and cross-state decoding accuracy for split s.s. (**B**) and consensus s.s. (**C**)

datasets in each sound category. Cross state decoding is shown for Wake only in **A**, **B**, and **C**. For all categories and both datasets, classification is significantly smaller for cross-state decoding than same-state decoding ($p < 0.05$, Wilcoxon signed-rank test). **D-F.** Sound population vectors for both states in the space of the first two principal components for Split s.s. (**D**) and Consensus s.s. (**F**) datasets The line represents the decision boundary line of the support vector machine classifier that discriminates both states. **E-G.** Regression plots of sound population vectors values for Wake and Anesthesia presentations in the rotated principal component space for Split s.s. (**E**) and Consensus s.s. (**G**) datasets. For each sound, a pair of blue (Wake) and red (Anesthesia) points shown in **D-F** can be plotted in the state space for both the dimension normal (top) or collinear (bottom) to the support classification line (see example sounds with highlighted markers).
